# Organelle reorganization in bovine oocytes during dominant follicle growth and regression

**DOI:** 10.1186/s12958-015-0122-0

**Published:** 2015-11-14

**Authors:** D. Dadarwal, G. P. Adams, P. Hyttel, G. M. Brogliatti, S. Caldwell, Jaswant Singh

**Affiliations:** Department of Veterinary Biomedical Sciences, Western College of Veterinary Medicine, University of Saskatchewan, 52 Campus Drive, Saskatoon, SK S7N 5B4 Canada; Department of Veterinary Clinical and Animal Sciences, University of Copenhagen, Groennegaardsvej 7, DK-1870 Frederiksberg C, Denmark; Universidad Católica de Córdoba, Reproduccion animal, Cordoba, X5000IYG Argentina

**Keywords:** Ultrastructure, Cattle, Cytoplasmic maturation, Mitochondria, Lipid, Stereology

## Abstract

**Background:**

We tested the hypothesis that organelles in bovine oocytes undergo changes in number and spatial distribution in a manner specific for phase of follicle development.

**Methods:**

Cumulus-oocyte-complexes were collected from Hereford heifers by ultrasound-guided follicle aspiration from dominant follicles in the growing phase (*n* = 5; Day 0 = ovulation), static phase (*n* = 5), regressing phase (*n* = 7) of Wave 1 and from preovulatory follicles (*n* = 5). Oocytes were processed and transmission electron micrographs of ooplasm representing peripheral, perinuclear and central regions were evaluated using standard stereological methods.

**Results:**

The number of mitochondria and volume occupied by lipid droplets was higher (*P* < 0.03) in oocytes from regressing follicles (193.0 ± 10.4/1000 μm^3^ and 3.5 ± 0.7 %) than growing and preovulatory stages (118.7 ± 14.4/1000 μm^3^ and 1.1 ± 0.3 %; 150.5 ± 28.7/1000 μm^3^ and 1.6 ± 0.2 %, respectively). Oocytes from growing, static and preovulatory follicles had >70 % mitochondria in the peripheral regions whereas oocytes from regressing follicles had an even distribution. Oocytes from growing follicles had more lipid droplets in peripheral region than in central region (86.9 vs. 13.1 %). Percent surface area of mitochondria in contact with lipid droplets increased from growing (2.3 %) to static, regressing or preovulatory follicle stage (8.9, 6.1 and 6.2 %). The amount, size and distribution of other organelles did not differ among phases (*P* > 0.11).

**Conclusions:**

Our hypothesis was supported in that mitochondrial number increased and translocation occurred from a peripheral to an even distribution as follicles entered the regressing phase. In addition, lipid droplets underwent spatial reorganization from a peripheral to an even distribution during the growing phase and mitochondria-lipid contact area increased with follicle maturation.

## Background

Oocyte quality determines the success of fertilization and subsequent embryonic development. Bovine oocytes achieve the competence to sustain fertilization and initial embryonic development to the blastocyst stage by the time the surrounding follicles reach 2–3 mm [1, 2]. The proportion of oocytes that developed to the blastocyst stage was higher for oocytes from follicles >6 mm compared to those from 2 to 6 mm follicles [2]. Similarly, higher blastocyst rates were observed for oocytes obtained from follicles >13 mm compared to those from 5 to 8 mm follicles [3]. In vitro blastocyst rates are reportedly higher for oocytes recovered from well defined static phase compared to the growing and regressing phase of dominant follicle development [4]. Furthermore, higher proportions of blastocyst were reported from in vitro fertilization (IVF) of in vivo matured oocytes (obtained after LH surge) than those matured in-vitro [5, 6]. The follicles (3–8 mm) that were aspirated for in vitro maturation had grown for short duration (1–4 days) compared to follicles that were aspirated after LH surge (6–9 days) [5, 6]. Above-mentioned findings suggest that oocytes undergo maturational changes as the follicle grows. The transcriptional activity of the oocyte is reduced to a minimum by the time a follicle reaches 3 mm in diameter (Fair et al., 1995, 1996), i.e., the follicle stage at the beginning of a follicular wave [7, 8]. Therefore, oocyte processes most likely influenced during follicular selection and dominance are cytoplasmic events such as changes to metabolic pathways, organelle structure and function, and their interactions with each other.

Among cytoplasmic organelles considered crucial for acquisition of oocyte competence, mitochondria and lipid droplets are critical for energy (ATP) production [9]. Moreover, mitochondria and the smooth endoplasmic reticulum (SER) are the primary source of Ca^2+^ oscillations that govern events such as nuclear maturation, fertilization and activation of embryonic development [10, 11]. The events of nuclear maturation following the preovulatory LH surge have been well characterized by transmission electron microscopy [12–15]. After resumption of meiosis, the majority of the oocytes reach metaphase II at 19–22 h after LH surge [12]. The events of cytoplasmic development before the LH surge during follicular dominance, however, are less studied. The results of an electron microscopy-based qualitative study suggest that structural changes with respect to different organelles occur in the dominant follicle before the LH surge [12].

Therefore, the objective of the present study was to evaluate structural changes in oocytes obtained from phase-specific dominant follicles by using objective quantitative criteria. Specifically we intended to characterize, quantitatively, the changes in numbers and distribution of mitochondria, lipid droplets, SER within the oocyte at different stages of dominant follicle growth and maturation. We tested the hypothesis that the number and spatial distribution of organelles within the ooplasm change in a phase-specific manner.

## Methods

### Collection of cumulus-oocyte-complexes

Twenty-two crossbred Hereford heifers were maintained in corrals at the Goodale Research Farm, University of Saskatchewan (Saskatoon, Canada). The heifers were between 14 and 22 months of age and had free access to hay, minerals and water. The University Committee on Animal Care and Supply approved the experimental protocol, and the study was conducted in accordance with the guidelines of the Canadian Council on Animal Care.

**Fig. 1:**
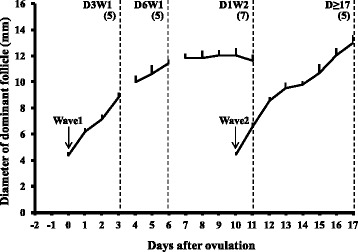
Diameter profiles (Mean + SEM, mm) of dominant follicles of Wave 1 (anovulatory) and Wave 2 (ovulatory). Mean days of follicular aspirations during different phases of development are indicated by dotted vertical lines: Day 3 of Wave 1 (D3W1), Day 6 of Wave 1 (D6W1), Day 1 of Wave 2 (D1W2) and preovulatory during estrus (Day ≥17). Numbers in parentheses show the number of oocytes examined for each stage of dominant follicular growth. Day 0 = emergence of wave 1

The ovarian follicular development was monitored daily by transrectal ultrasonography (Aloka SSD-500 echocamera with 7.5 MHz linear array transducer, UST 5821–7.5 Aloka Co. Ltd, Tokyo, Japan). The location and antral diameter of individual follicles (>4 mm) in both ovaries were recorded on data sheets (Knopf et al. 1989). Following spontaneous ovulation (Day 0), heifers were assigned randomly for COC collection from dominant follicles (Fig. 1) on Day 3 to 4 (growing phase of the Wave 1 dominant follicle, D3W1, *n* = 5), Day 6 to 7 (static phase of the Wave 1 dominant follicle, D6W1, *n* = 5), one day after the emergence of second follicular wave (Days 10 to 12; regressing phase of the Wave 1 dominant follicle, D1W2, *n* = 7), and on Days ≥17 (preovulatory follicle after detection of signs of estrus, D17, *n* = 5). Only one follicle from each animal was aspirated and none of the animal was repeated for COC collection.

The COCs were collected using a modified 5 MHz end-fire transvaginal transducer attached to Aloka 500 echocamera as described previously (Brogliatti and Adams 1996). The follicles were aspirated using an 18 gauge, 60 cm long, single-lumen needle with the flow rate of 30 ml/min (Vacuum pump: Allied Healthcare Products, Inc, St Louis, MO, USA). Follicular contents were collected in 15 ml centrifugation tubes containing 3 ml phosphate buffer saline (PBS) with bovine serum albumin (0.2 %) as surfactant and sodium heparin (10 IU/ml) as anticoagulant. The COCs were searched under stereozoom microscope and fixed in 1 % gluteraldehyde and 0.1 M sodium cocodylate buffer (pH 7.4) and stored at 4 °C until further processing for transmission electron microscopy.

### Processing of oocytes for transmission electron microscopy

**Fig. 2:**
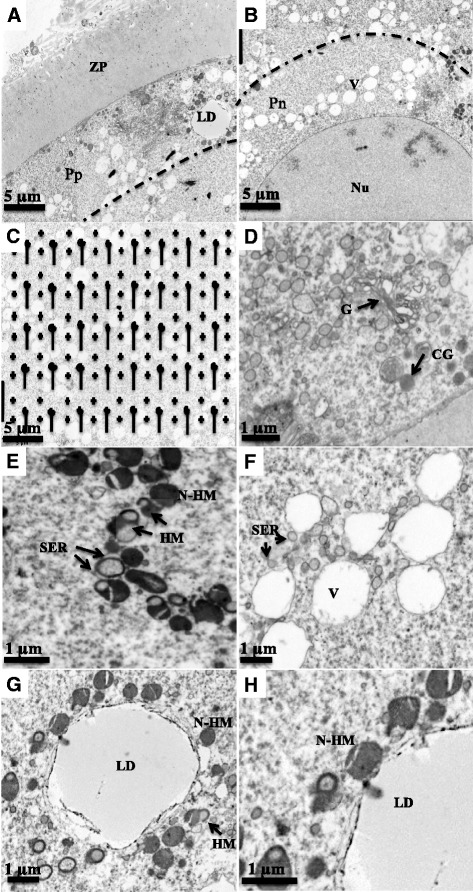
Electron micrographs (photographed at a primary magnification of 3000x) representing peripheral (Pp, **a**), perinuclear (Pn, **b**), and central (**c**) regions of ooplasm as well as the central region overlayed with the transparent grid for stereology (**c**). **d**, **e**, **f**, **g**, **h** illustrate cortical granules (CG), Golgi complex (G), hooded (HM) and non-hooded mitochondria (N-HM), vesicles (V), smooth endoplasmic reticulum (SER), and lipid droplets (LD) identified in the ooplasm for the quantitative data. Note the close spatial association between hooded-mitochondria (HM) and SER (**e**); and non-hooded mitochondria (N-HM) and lipid droplet (**h**). **d**, **e**, **f** and **g** represents peripheral ooplasm and (**h**) represent perinuclear ooplasm of an oocyte from D6W1 follicle, respectively. ZP = zona pellucida. Nu = nucleus

The COCs were stained with 1 % Toluidine blue and placed into liquid 1 % agarose at 40°-42 °C (Sigma type II, A 6877) and allowed to cool into blocks at room temperature. The COC-agarose blocks were washed three times with 0.1 M sodium cocodylate and post-fixed with fresh 1 % osmium tetraoxide (in sodium bicarbonate buffer, pH 7.4) for 1 h at room temperature. The COC-agarose blocks were then dehydrated in 50 % ethyl alcohol (5 min) and stained (1 h) with saturated uranyl acetate in 70 % ethyl alcohol. Further dehydration was done by sequential treatment of the tissues in 70, 95, and 100 % ethyl alcohol (5 min each). The blocks were then washed with propylene oxide (three times, 5 min each). The blocks were incubated in 2:1 and 1:2 mixture of embedding media (epon/araldite:propylene oxide) for 30 min and two hours, respectively, and were then left overnight in pure epon/araldite embedding media. The COC-agarose blocks were then placed in flat embedding molds to polymerize in embedding medium at 60 °C for 48 h. The COCs were sectioned serially using ultramicrotome (Ultratome III, Catalogue # 8801A, LKB, Stockholm, Sweden) at a thickness of 0.5-1 micron. In addition, ultrathin sections (60–80 nm) were obtained at the largest diameter of COC and at the level of the nucleus. Ultrathin sections were collected on 75 x 300 mesh copper grid. Electron micrographs of peripheral ooplasm (ooplasm area within 10 μm of the oocyte plasma membrane), perinuclear ooplasm (area within 10 μm of the nuclear envelope) and central ooplasm (area excluding peripheral and perinuclear regions) (Fig. 2) of an oocyte were taken at 3000x primary magnification using a Philips 410LS transmission electron microscope. Negatives were printed on 11 inch x 14 inch photographic paper (Kodachrome II RC, Catalogue # 1922970, Eastman Kodak Co., Rochester, NY) to obtain a final magnification of 10,000x.

### Analyses of cytoplasmic organelle parameters

Quantitative analyses of areas representing peripheral, perinuclear and central ooplasm regions were performed by standard stereological methods that involved random placement of a transparent test-grid (Fig. 2c) over an electron micrograph [16]. The distance between each adjacent cross-points on the grid was 1.5 cm and line lengths were 1.5 cm; therefore the area associated with each cross-point was 2.25 cm^2^. Peripheral and perinuclear 10 μm regions were identified and demarcated on respective electron micrographs considering that a 1 cm distance on the electron micrograph represented a 1 μm distance within the oocyte (calculated from magnification achieved at printing the electron micrographs). The surface area density (surface area of organelle per unit volume of cytoplasm; μm^2^/μm^3^), volume density (volume of organelle per unit volume of cytoplasm; μm^3^/μm^3^) and numerical density (number of organelle per unit volume of cytoplasm; number/μm^3^) were calculated for each organelle [17, 18]. For simplicity, the volume density and numerical density of organelles is presented as percent and number/1000 μm^3^ of oocyte volume. The points where the lines of the overlay grid intersected the contact between two organelles were used to calculate the contact area and are presented as percent area of mitochondria.

### Statistical analyses

All statistical analyses were done using SAS 9.2 (SAS Institute Inc., Cary, NC, USA). Comparisons among stages of dominant follicle growth were made by one-way analysis of variance. When the regions were compared across the stages of follicle growth, two-way factorial analysis of variance was used. Proportional data were transformed to the arcsine and compared among stages by analysis of variance. The least significant difference was used as a post-hoc test. Data are presented as mean ± standard error (SE) and a *P*-value ≤0.05 was considered significant. The data for central and perinuclear regions were pooled for the endpoints where significant differences were not observed. The pooled data for central and perinuclear regions was compared with the data for peripheral region for respective endpoints.

## Results

### Qualitative morphological observations

The oocytes presented a series of typical membrane-bounded organelles (mitochondria, vesicles, SER, Golgi complexes and cortical granules) and lipid droplets (Fig. 2). The mitochondria exhibited two morphologies, hooded and non-hooded (Fig. 2e). Both morphologies were closely associated with SER (Fig. 2f) and lipid droplets (Fig. 2h) as clusters. The vesicles coalesced and became larger as the follicles progressed towards regression.

### Mitochondria

**Fig. 3:**
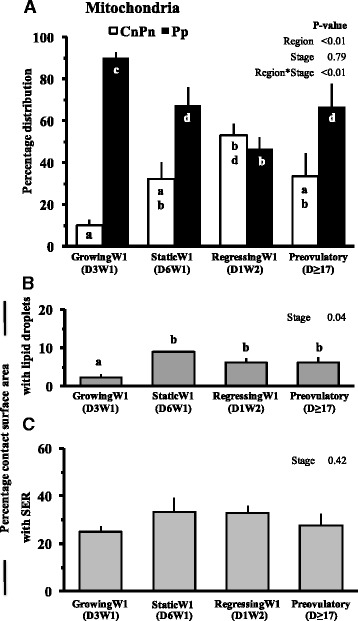
Mean ± SE of percent distribution of mitochondria in different regions of ooplasm (**a**), percentage of mitochondrial surface in contact with lipid droplets (**b**), and smooth endoplasmic reticulum (**c**) in oocytes collected at different stages of follicular growth and maturation: Day 3 of Wave 1 (D3W1), Day 6 of Wave 1 (D6W1), Day 1 of Wave 2 (D1W2) and preovulatory during estrus (Day ≥17). Pp and CnPn represent peripheral and central plus perinuclear regions of oocyte, respectively. ^ab^Values with no common superscript are different (*P* < 0.05)

**Fig. 4:**
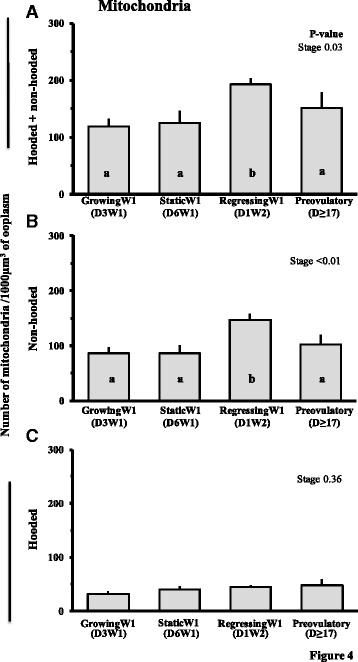
Number (Mean ± SE) of total (non-hooded and hooded combined) (**a**), non-hooded (**b**) and hooded (**c**) mitochondria per 1000 μm^3^ of oocyte from follicles at different stages of follicular growth and maturation: Day 3 of Wave 1 (D3W1), Day 6 of Wave 1 (D6W1), Day 1 of Wave 2 (D1W2) and preovulatory during estrus (Day ≥17). ^ab^Values with no common letters are different (*P* < 0.05)

A significant majority (>70 %) of the mitochondria were located in the peripheral regions of the oocytes from growing, static and preovulatory follicles. However, mitochondria were evenly distributed across the peripheral and central/perinuclear regions of oocytes from regressing follicles (Fig. 3a) because each region presented approximately 50 % of the total mitochondrial population. Volume of the mitochondrial compartment ranged from 1 to 4 % of total ooplasmic volume depending on the stage of follicular growth (Table 1). The mitochondrial volume and surface densities was higher (*P* = 0.04) in oocytes from regressing follicles compared to oocytes from follicles at other stages of follicle development (Table 1). The mitochondrial numerical density (mitochondria per 1000 μm^3^ volume of ooplasm) was highest (*P* = 0.03) in oocytes from regressing follicles; about 63, 54 and 28 % more than in the oocytes from growing, static and preovulatory stage follicles, respectively (Fig. 4a). The numerical density of non-hooded mitochondria changed (Fig. 4b) but hooded mitochondria numerical densities remained unchanged (Fig. 4c) with the follicular status studied. However, distribution of hooded mitochondria changed across follicular status studied. Higher number of (*P* < 0.01) hooded mitochondria were located in the peripheral region of oocytes from growing follicles compared to oocytes from static, regressing phase and preovulatory follicles (95.3 ± 6.3/1000 μm^3^ vs. 69.1 ± 1.2/1000 μm^3^, 50.7 ± 6.8/1000 μm^3^ and 76.8 ± 15.2/1000 μm^3^, respectively).

**Table 1:** Volume (percent of ooplasm) and surface density (Mean ± SEM, μm^2^/μm^3^ of ooplasm) of mitochondria, SER profiles and vesicles; and number (Mean ± SEM, per 1000 μm^3^ of ooplasm) of SER profiles and vesicles in oocytes at different stages of follicular development

Organelle	Parameter (*P*-value)	GrowingW1 (D3W1)	StaticW1 (D6W1)	RegressionW1 (D1W2)	Preovulatory (D ≥ 17)
Mitochondria	Volume (%) (*P* = 0.04)	2.3 ± 0.2^a^	2.6 ± 0.3^a^	3.9 ± 0.5^b^	2.4 ± 0.4^a^
	Surface Density (μm^2^/μm^3^) (*P* = 0.03)	0.287 ± 0.035^a^	0.299 ± 0.046^a^	0.451 ± 0.039^b^	0.326 ± 0.049^a^
SER profiles	Volume (%) (*P* = 0.87)	3.9 ± 0.2	4.9 ± 0.1	4.7 ± 0.8	4.4 ± 0.4
	Surface Density (μm^2^/μm^3^) (*P* = 0.34)	0.652 ± 0.065	0.599 ± 0.106	0.780 ± 0.065	0.653 ± 0.055
	Number (/1000 μm^3^) (*P* = 0.78)	521.5 ± 87.5	467.3 ± 84.9	670.1 ± 85.4	650.2 ± 40.9
Vesicles	Volume (%) (*P* = 0.42)	20.4 ± 2.9	16.4 ± 1.7	16.2 ± 2.8	21.6 ± 3.2
	Surface Density (μm^2^/μm^3^) (*P* = 0.84)	0.972 ± 0.126	0.878 ± 0.073	0.884 ± 0.141	1.005 ± 0.083
	Number (/1000 μm^3^) (*P* = 0.86)	197.1 ± 33.3	185.8 ± 23.7	254.3 ± 94.4	202.4 ± 9.3

^ab^Values with different superscripts are different (*P* < 0.05). Analyses were done on proportions, not percentages

### Mitochondrial relation with lipid droplets and SER profiles

The percent surface area of mitochondria in contact with lipid droplets was less (*P* = 0.04) in the oocytes from growing stage follicles compared to other stages but was not significantly different between other stages (Fig. 3b). In all regions collectively, the percent surface area of mitochondria in contact with SER profiles did not vary (*P* = 0.42) with the phases of follicle growth (Fig. 3c).

### Lipid droplets

**Fig. 5:**
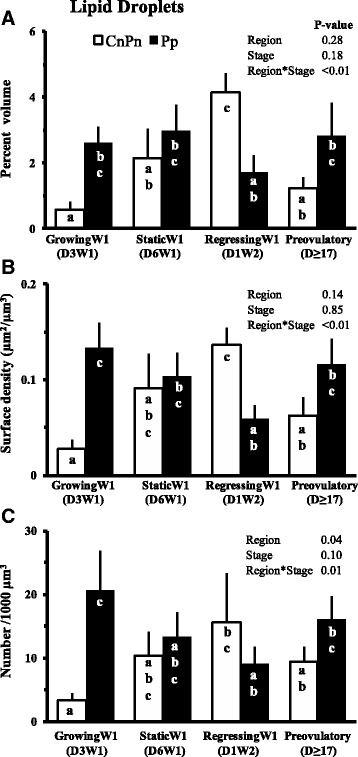
Mean ± SE of percent volume (**a**), surface area (**b**, μm^2^/μm^3^ of oocyte) and number (**c**, per 1000 μm^3^ of oocyte) of lipid droplets. Pp and CnPn bars represent peripheral and central plus perinuclear regions of oocyte, respectively. ^ab^Values with no common letters are different (*P* < 0.05)

The changes in lipid droplet volume, surface and numerical densities in different regions of oocyte from follicles at different phases of follicular development are shown in Fig. 5. In all regions taken together, lipid droplets accounted for 1 to 5 % of ooplasmic volume. The lipid droplet volume was less (*P* < 0.01) in oocytes from growing and preovulatory phase follicles (1.1 ± 0.3 % and 1.6 ± 0.2 %, respectively, Fig. 5a), it increased to 2.3 ± 0.5 % in the static phase and was highest in oocytes from regressing phase follicles (3.5 ± 0.7 %). The overall surface density of lipid droplets in oocyte tended to differ (*P* = 0.09) with the stage of follicular maturation (data not shown), but no change was detected in the overall number of lipid droplets (/1000 μm^3^ of ooplasm). The peripheral region of oocytes from growing phase follicles had approximately 5 times more number of lipid droplets than the central/perinuclear regions (region *P* = 0.04, stage *P* = 0.10 and region*stage *P* = 0.01, Fig. 5c). During later stages of follicular development, the number of lipid droplets was not different between peripheral vs. central/perinuclear regions of the ooplasm.

### Other organelles

**Fig. 6:**
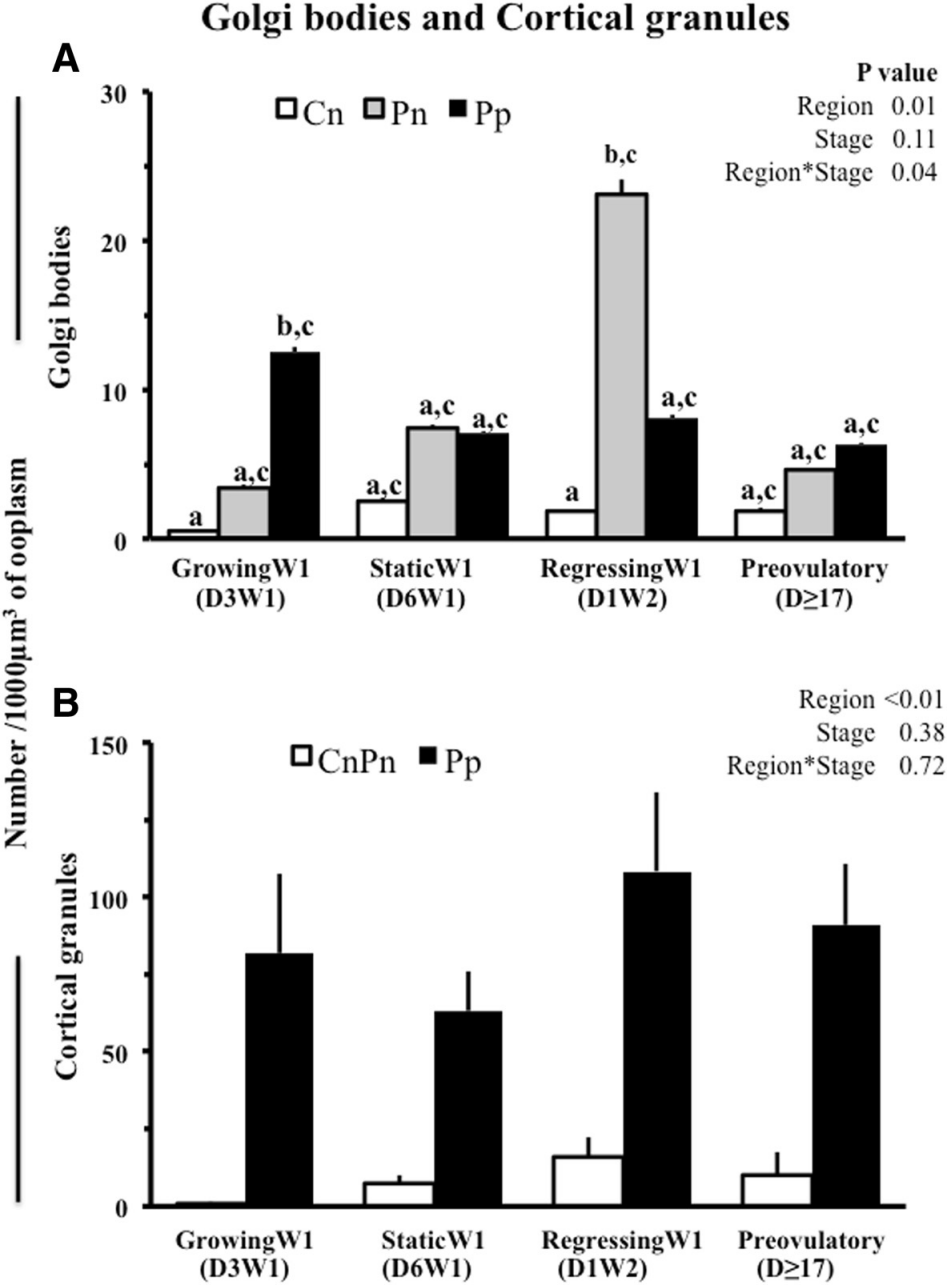
Mean ± SE of number of Golgi complexes (**a**) and cortical granules (**b**) in 1000 μm^3^ of oocyte from follicles at different stages of follicular growth and maturation: Day 3 of Wave 1 (D3W1), Day 6 of Wave 1 (D6W1), Day 1 of Wave 2 (D1W2) and preovulatory during estrus (Day ≥17). Cn, Pn‚ Pp and CnPn represent central, perinuclear‚ peripheral regions and central plus perinuclear regions of oocyte, respectively. ^ab^Values with no common letters are different (*P* < 0.05)

The vesicles were the most abundant structure in the oocyte; their volume ranged from 7 to 31 % of ooplasmic volume. However, no significant changes were observed in the volume, surface and numerical densities of the vesicles across different phases of follicular development (Table 1). The SER profiles accounted for 2 to 10 % of ooplasmic volume (Table 1). The volume, surface and numerical densities of SER profiles in oocytes did not change significantly with the phase of follicular development (Table 1). There were no significant changes in the number of Golgi complexes when all regions taken together were compared across different phases of follicular maturation. When regions were compared between and within different stages, the peripheral region of oocytes from growing follicle had three times as many Golgi complexes as the perinuclear regions (Fig. 6a), whereas, the perinuclear region of oocytes from regression phase follicles had twice the number of Golgi complexes than the peripheral region. More than 87 % of cortical granules were located in the peripheral region of the oocytes and their distribution did not change significantly with the phase of follicular development (Fig. 6b). Likewise, the number of cortical granules did not change across the follicular stages.

## Discussion

**Fig. 7:**
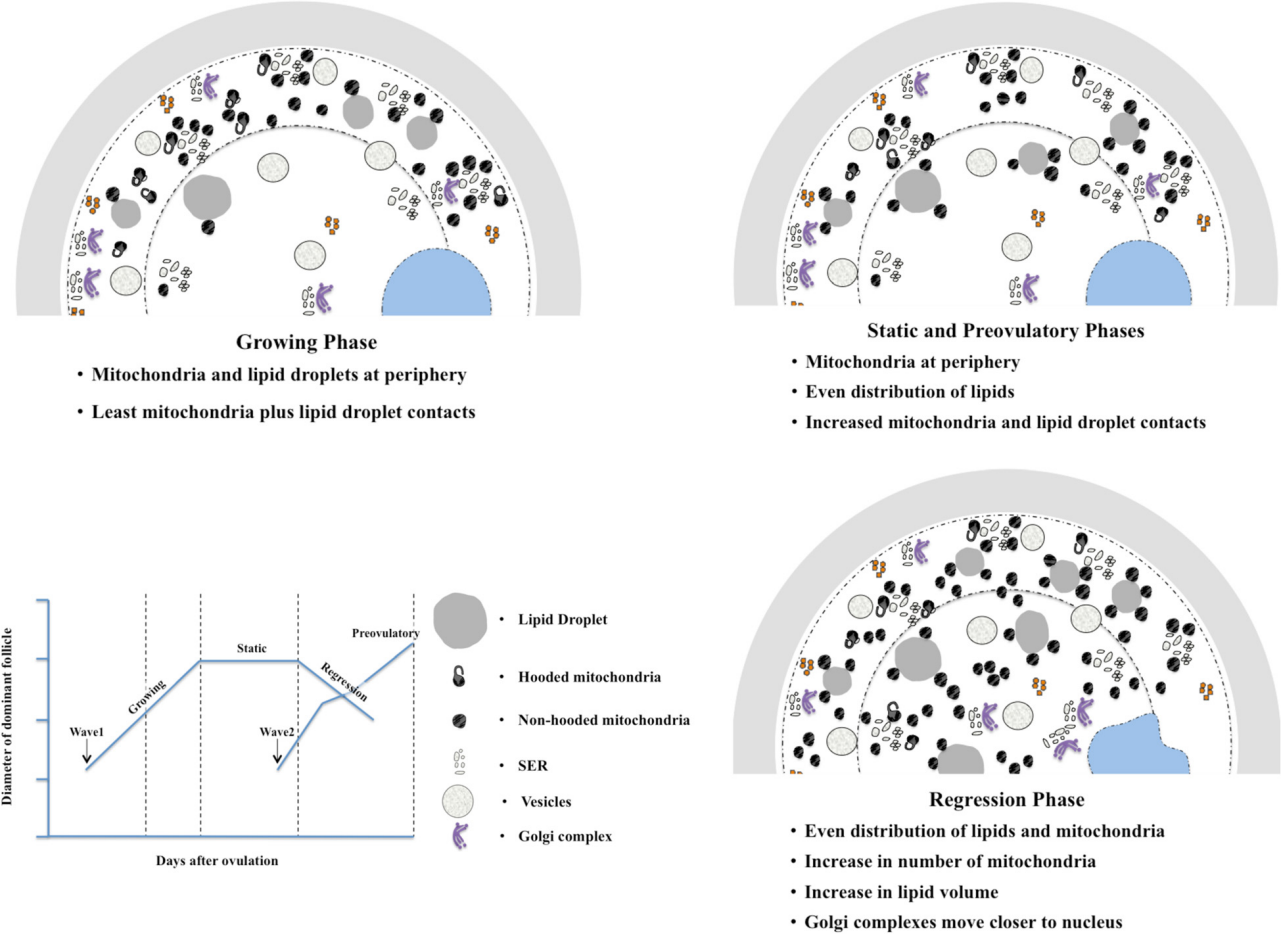
Schematic summary of the changes in organelle characteristics within the oocytes during the dominant follicle development

In the present study, the hypothesis that the number and spatial distribution of organelles within the ooplasm changes in a phase-specific manner during dominant follicle development was supported. The major changes in organelle numbers, spatial distribution and their interaction are summarized in Fig. 7. The number of mitochondria increased and changed from a peripheral to an even distribution in the oocyte as the dominant follicle entered the regressing phase. Similarly, lipid droplets changed from a peripheral location in oocytes of growing-phase dominant follicles to an even distribution in static-phase dominant follicles, and assumed an even more central location in the regressing phase. Although the number of lipid droplets did not change, the percent volume occupied by lipid droplets (i.e., amount) increased in the regressing phase.

Importantly, the COC in the present study were collected at well-defined phases of dominant follicle development. Oocyte diameter in cattle reaches a plateau by the time antral follicles reach a diameter of 3 mm [19] but distinct changes in form and function of antral follicles, and presumably the contained oocyte, occur after this developmental stage. The development of ovarian follicles ≥1 mm has been well-characterized using transrectal ultrasonography [20–22] and occurs in a two- or three-wave pattern during the intervoulatory interval [23, 24] Each wave is initiated by a surge in circulating FSH [25], and the follicles of the cohort grow with equal pace for 2–3 days when all but one follicle begins to slow. At this time of selection, the dominant follicle is around 8.5 mm in size [7] and attains ovulatory capacity as it acquires LH receptors while other follicles fail to do so [26]. In the presence of a functional corpus luteum and basal circulating LH concentrations, the dominant follicle continues to grow for another 2–3 days and then enters a static phase [7]. After a static phase of about three days, the next follicular wave emerges and the dominant follicle of the previous wave begins a regressing phase. During luteolysis, LH pulsatility increases and the extant dominant follicle continues to grow until the time of ovulation after the LH surge [27, 28]. The competence of the contained oocyte to develop into an embryo increases as the follicle grows [2, 3] and peaks during the late static phase of follicle development [4]. The basis of this increase in oocyte competence has not been fully elucidated, but may be related to changes in cytoplasmic organelle morphology and distribution [12].

Mitochondria remained in the peripheral ooplasm of oocytes from dominant follicles in the growing, static and preovulatory phases. In previous studies, an even distribution of mitochondria was observed in bovine oocytes of preovulatory follicles ≥19 h after LH surge, when the majority of oocytes had reached MII [12, 15]. None of the oocytes from preovulatory follicles in the present study were at this late stage of meiosis, and we did not observe a translocation of mitochondria to a more even distribution. Interestingly, we noted this even distribution of mitochondria in oocytes from regressing-phase dominant follicles (Fig. 7). As previously described [12], oocytes from regressing follicles appear to display structural features similar to that of preovulatory oocytes collected after the LH surge. In the present study, two of seven oocytes from regressing follicles exhibited undulations of the nuclear envelop suggesting activation of meiosis. Similar findings were reported elsewhere for the regressing dominant [12] and subordinate follicles [29], raising the question about whether the LH surge is requisite for resumption of meiosis. The LH surge has been associated with a build-up of reactive oxygen species (ROS) in the preovulatory follicle, which is required for cumulus cell expansion and ovulation [30]. We speculate that regressing follicles that are undergoing atresia [31] have increased production of ROS that simulates maturational changes induced by LH in the preovulatory follicle. This may explain why oocytes collected from early regressing follicles may be efficient in sustaining embryonic development upon IVM and IVF [4].

The increase in the number of mitochondria in our study is attributed specifically to an increase in the number of non-hooded mitochondria. In contrast to results of an earlier report [32] no changes were detected in the number of hooded mitochondria in the present study. It should be noted, however, that examination of ultra-thin sections would result in an over-estimation of non-hooded mitochondria as hooded mitochondria will appear non-hooded in many sections. The specific roles of the two types of mitochondria are unknown, but some suggest that hooded mitochondria are immature forms that limit the production of reactive oxygen species in the oocyte during maturation and embryonic development (Crocco 2011, Van Blerkom 2004). Others suggest that the hooded morphology increases the functional surface area by extending both outer and inner mitochondrial membranes [33]. Similar to our observations, the hooded type often encircle SER which are crucial for exchange of Ca^2+^ needed for variety of cell signaling [34].

Lipid droplets store triglycerides as a source of ATP needed for oocyte maturation and further embryonic development [35]. A previous report indicated an increase in lipid droplet volume as the follicle grow after LH surge and the oocyte progress towards its final maturation [15]. In the present study, an increased lipid content was obvious only when the follicles entered the regressing phase. It is not known why atresia leads to an increased lipid droplet volume per unit of ooplasm in the present study. Interestingly, we have observed bigger lipid droplets in poor quality oocytes obtained from follicles undergoing atresia following superstimulation with FSH starvation [36]. Previously an increase in lipid droplet volume density has been suggested due to an increase in hooded mitochondria that fail to utilize lipid [37]. However, we did not observe any change in the number of hooded mitochondria despite an increase in lipid droplet volume in oocytes as follicles progressed from growing to regressing stages. A close spatial relationship between mitochondria and lipid droplets in oocytes has been reported in cattle [12, 15] and other species [38]. Fluorescence resonance energy transfer analyses of stained mitochondria and lipid droplets in porcine oocytes showed that these two organelles are located within ≤ 10 nm distance to each other that has been considered adequate [35] for generation of ATP through β-oxidation of lipids, a process that requires carnitine [9]. We speculate that close proximity between mitochondria and lipids during static, regressing and preovulatory phases of dominant follicle development may reflect a greater dependence of the oocytes in these follicles on lipid-derived ATP generation for energy requirements.

Our findings supported previous observation [12, 15] that SER associated vesicles are the most abundant organelle in the oocytes. However, no change in size, number or distribution was observed in the oocytes from follicles of different phases of development in the present study.

Golgi complexes translocate within cytoplasm along microtubules [39] and are involved in the transport, processing and secretion of proteins, and in the processing of macromolecules following endocytosis [40, 41]. In a recent study of bovine oocytes [39], these organelles moved from the central to the peripheral ooplasm during germinal vesicle breakdown (GVBD) and back to a central location at the MI stage. In the present study, however, Golgi complexes were located peripherally during the growing phase and centrally during the regressing phase. In the preovulatory follicles, evenly distributed Golgi complexes were (Fig. 7) in accordance with a central movement of these organelles during final oocyte maturation, which in our study did not include GVBD. The difference between studies may be attributed to differences between in vitro maturation conditions [39] vs. in vivo conditions (present study).

Golgi-derived cortical granules contain the recently identified protease, Ovastacin, which is responsible for cleavage of zona pellucida protein-2 (ZP-2) and preventing polyspermy following fertilization [42, 43]. In previous reports, cortical granules were observed to translocate along microfilaments [44] from cortical clusters to solitary locations along the oolemma late during final bovine oocyte maturation [13, 15]. In the present study, cortical granules were located peripherally in all phase follicles, but appeared prevalent in oocytes from preovulatory and regressing phase follicles.

Based on the mitochondrial distribution and lipid-mitochondrial contacts it appears that oocytes from growing phase dominant follicles have not yet fully completed the cytoplasmic maturation. At this stage, these oocytes were days away from atresia or ovulation and perhaps have least dependency on ooplasmic lipids for energy demands. We postulate that the oocytes from static phase follicle were morphologically similar to those from preovulatory phase follicles as they had mitochondria in the peripheral region and increased contacts between mitochondrial and lipid droplet compared to other phases. Both static and preovulatory phase follicles have undergone similar period of growth after wave emergence thus it is expected that the cytoplasmic maturation of the contained oocyte will be nearing completion for nuclear maturation and subsequent fertilization, both of which are high energy dependent events. Regression phase follicles have oocytes that has started to undergo atresia (due to loss of dominance) and thus would potentially have less ATP and more ROS generating mitochondria. We hypothesize that mitochondria and lipid droplets redistribution is associated with oocyte’s final attempt to survive. Conversely, cytoplasmic changes observed in the oocytes from early regressing follicles may be reflective of enhanced developmental competence as suggested by earlier studies [12, 29]. Future studies should focus on understanding proposed functional aspects of different organelles (especially mitochondria and lipids) in oocytes from follicles at different phases of follicle development. Such studies would potentially help redesign in vitro embryo production methodology where heterogenous population of follicles is aspirated to obtain oocytes.

## Conclusions

We conclude that organelle numbers and distribution patterns occur in a manner specific to the phase of follicular growth, maturation and regression (Fig. 7). Oocytes from follicles in the growing phase displayed the least area of mitochondrial contact with lipid droplets, and a peripheral distribution of lipids. Oocytes from static phase follicle were similar to those from preovulatory phase follicles as they had mitochondria in the peripheral region and increased contacts between mitochondrial and lipid droplet compared to other phases. Oocytes from regressing phase follicles were characterized by an increase in mitochondrial number, and an even distribution of mitochondria. Oocytes from follicles in the regressing phase had greater percent lipid volume than any other phase.
